# Changes in Medical Student and Doctor Attitudes Toward Older Adults After an Intervention: A Systematic Review

**DOI:** 10.1111/jgs.12312

**Published:** 2013-06-10

**Authors:** Rajvinder Samra, Amanda Griffiths, Tom Cox, Simon Conroy, Alec Knight

**Affiliations:** * Institute of Work, Health and Organisations, University of NottinghamNottingham, UK; † School of Business, Economics and Informatics, Birkbeck, University of LondonLondon, UK; ‡ Department of Cardiovascular Sciences, University of LeicesterLeicester, UK; § Work Psychology GroupDerby, UK

**Keywords:** doctor, medical student, attitude, intervention, older adult

## Abstract

Research investigating the effects of attitude-focused interventions on doctors’ and medical students’ attitudes toward older adults has produced mixed results. The objective of this systematic review was to determine whether factors pertaining to study design and quality might provide some explanation of this inconclusive picture. Articles were judged of interest if they reported doctors’ or medicals students’ attitude scores before and after a geriatric-focused intervention. Articles that did not report the measure used, mean scores, or inferential statistics were excluded. Twenty-seven databases, including Medline, PsychInfo, and Embase, were searched through April 2011 using a systematic search strategy. After assessment and extraction, 27 studies met the eligibility criteria for this review. These studies demonstrated inconsistent results; 14 appeared successful in effecting positive attitude change toward older adults after an intervention, and 13 appeared unsuccessful. Attitude change results differed in line with the content of the intervention. Of the 27 studies, 11 interventions contained solely knowledge-building content. Three of these studies demonstrated positive changes in doctors’ or medical students’ attitudes toward older adults after the intervention. The remaining 16 interventions incorporated an empathy-building component, such as an aging simulation exercise or contact with a healthy older adult. Of these, 11 successfully demonstrated positive attitude change after the intervention. The inclusion of an empathy-building task in an intervention appears to be associated with positive attitude change in medical students’ and doctors’ attitudes toward older adults.

The world’s population is living longer.^1^ Along with the many benefits of an older population, there exist some challenges in meeting the changing healthcare demands. In the United States, people aged 65 and older constitute 13% of the population and account for 37% of all hospital healthcare expenses.^2^ Doctors will see a large proportion of patients aged 65 and older throughout their working careers. Despite the increasing numbers of older adults in hospital settings, medical students have commonly reported little interest in caring for this patient group.^3^ A variety of interventions designed to prepare and encourage medical students to care for older adults have been developed. The interventions employed range from an educational course in geriatric care to mentoring with healthy older members of the community (henceforth, referred to as a “geriatrics intervention”). The outcomes of such interventions have typically been based on measures of attitudes toward, or knowledge about, older adults.

Interventions designed to improve knowledge about older adults are more successful than interventions designed to increase positive attitudes toward older adults.^4^ A systematic review of the effects of such interventions^4^ concluded that knowledge about older adults increased after a geriatrics intervention in nine of 12 studies that investigated knowledge change. Of the 19 studies investigating attitude change after a geriatrics intervention, 10 demonstrated positive attitude change, and nine demonstrated mixed results or no significant change. In short, although knowledge scores increased after a knowledge-directed geriatrics intervention, attitude scores did not change by significantly more than chance after an attitude-directed geriatrics intervention.

Although inconsistent results for attitude change after a geriatrics intervention were found, the disparate study designs employed, the type and duration of intervention, and the choice of questionnaire used to measure attitudes might explain this inconsistency.^4^ Following on from this, the present review makes use of these disparate study designs, as well as an examination of methodological quality, to identify study characteristics associated with positive attitude change. It aims to identify the conditions under which interventions are most effective in changing attitudes toward older adults.

## Objectives

A systematic review of the literature was conducted to determine the reported success or failure of training interventions to improve medical students’ and doctors’ attitudes toward older adults and whether study design and study quality were associated with change in attitude scores.

## Method

### Inclusion Criteria

- Medical students or medical doctors in secondary or tertiary care settings.
- Incorporates a geriatrics-focused intervention.
- Measure attitudes (e.g., cognition, intention to behave, stereotypes) toward adults aged 65 and older.
- Attitude scores collected before and after intervention.
- Published from database inception to April 30, 2011.
- Available in English.
- Published in a peer-reviewed journal.

### Exclusion Criteria

- Studies that include only primary care physicians.
- Studies that take place in community practice settings.
- Studies that do not relate to human subjects aged 18 and older.
- Studies that do not report the measure used or, in the case of locally developed measures, provide items employed.
- Studies that do not provide mean attitude pre- and postintervention scores.
- Studies that do not report *P*-values.
- Results duplicated in another study included in the review.

### Search Strategy

The search terms were: (physician [indexed term] or doctor or physician or consultant or registrar or clinician or hospitalist or internist or surgeon or geriatrician or psychogeriatrician or psychiatrist or cardiologist or gastroenterologist or neurologist or oncologist or respirologist or rheumatologist or dermatologist or urologist or endocrinologist or hepatologist or nephrologist or ophthalmologist or physiatrist or anesthesiologist or anaesthetist or pulmonologist or otolaryngologist or medical student or medical resident or medical fellow or medical professional or medical specialist or medical practitioner or medical officer or medical intern or medicine student or medicine resident or medicine fellow or medicine professional or medicine specialist or medicine practitioner or medicine officer or medicine intern or house officer or associate specialist) AND (aged [indexed term] or old* person or old* patient or old* adult or elder* or frail or ageing or aging or aged care or aged patient or aged person or geriatric care or geriatric patient or geriatric person or old age or seniors or senior citizen or senior adult or senior person or senior patient) AND (attitude [indexed term] or attitud* or belief or ageis* or agis* or discriminat* or prejudic* or preconception or misconception or stereotyp* or attribution or opinion or stigma or label?ing or age bias) [in title or abstract].

The following databases were searched using the following search strategy: ABI/Inform, Allied and Complementary Medicine Database (AMED), Applied Social Sciences Index and Abstracts, British Nursing Index, Business Source Premier, CAB Abstracts International, Cochrane Database of Systematic Reviews, CSA Sociological Abstracts, Cumulative Index to Nursing & Allied Health (CINAHL), Database of Abstracts of Reviews of Effects, Embase, Educational Resources Information Center (ERIC), Global Health Archive, Health Information Management Consortium, Health Technology Assessment, International Bibliography of the Social Sciences (IBSS), ISI Web of Science, Journal Storage (JSTOR), Medline, NHS Economic Evaluations Database, Politics and International Studies (PAIS International), PubMed, PyscInfo, SciVerse Scopus, Social Science Abstracts, SPORTDiscus, and Zetoc. The search produced 12,305 hits across all databases with duplicates removed.

### Initial Assessment of Relevance

**Figure 1:**
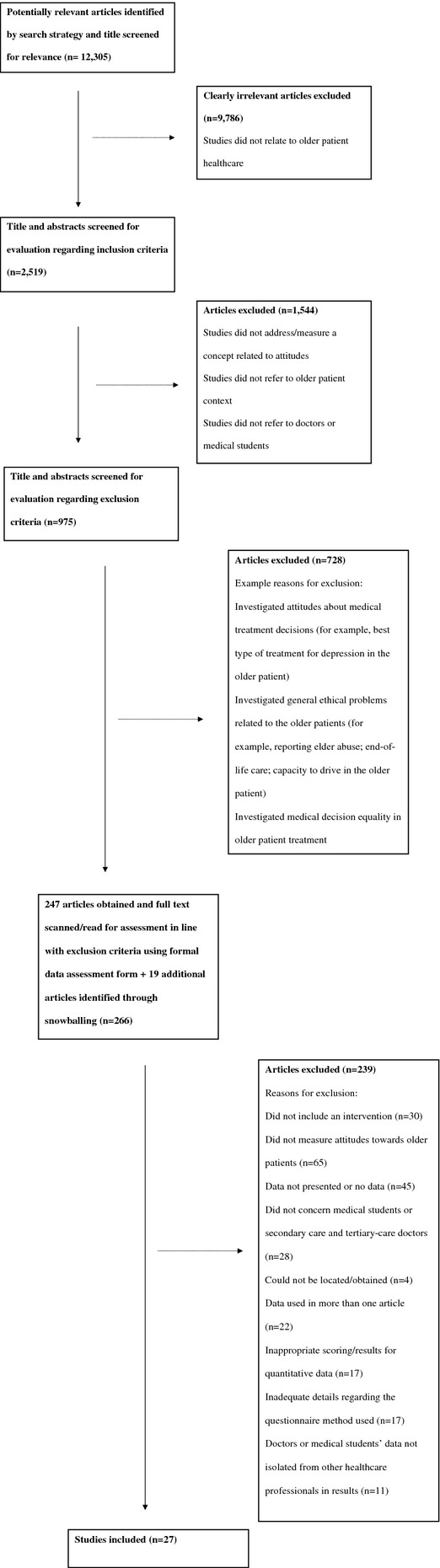
Flowchart of study inclusion and exclusion.

The titles and abstracts of the 12,305 search results were scanned to remove obviously irrelevant articles, leaving 2,519 articles. These abstracts were screened according to the eligibility criteria, removing a further 2,272 articles. The main reasons for exclusion were that studies did not measure attitudes, did not address older adults, or did not include medical students or doctors as the participant group. Figure 1 is a flowchart showing study exclusion. One reviewer (RS) made all decisions, but any uncertainties were discussed with another member of the research team (AG). Copies of the 247 articles were obtained and examined, and their reference lists were checked for potentially relevant articles that the search strategy had not identified, resulting in a further 19 potentially relevant articles. The full texts of the 266 articles were scanned to confirm that each met all of the eligibility criteria. Review of the full text removed a further 239 articles, with the main reasons for exclusion being an absence of a geriatrics intervention or no provision of attitude score data. A total of 27 articles met the inclusion criteria for the present review.

### Data Assessment and Extraction

The strengths and weaknesses of studies were assessed using an evaluation tool.^5^ Studies that had compromised methodology were identified during this quality assessment phase, but were nonetheless included in analyses to identify any methodological factors associated with positive attitude change. Justifications for the decision to code a study as methodologically weak are discussed in the Results section. All data assessed with the evaluation tool^5^ were extracted and comprised the following: bibliographic details of the study (author names and date published), setting (location of study), participant characteristics (age range, medical school year group or doctor grade), study design (study type, number of groups), intervention details (content of intervention, duration and frequency of exposure), comparison interventions (use of comparison group, use of alternative exposure), time period of measurement, sample selection (size of source group, selection method, random allocation, group size justification, comparability of groups), study method (attrition, control of confounders), study instruments (instrument used, outcome measurement criteria, validity and reliability reports, attitude scores), data analysis methods (suitability of statistical techniques), and process problems (reported problems in data collection).

### Data Analysis

Studies were categorized according to whether they demonstrated positive change in attitude (successful) or no positive change (unsuccessful) (see Table 1). For the purpose of this analysis, in studies without a comparison group, positive change was deemed to have occurred when postintervention scores were significantly higher than preintervention scores. In studies with a comparison group, significantly different postintervention attitude scores between the intervention and comparison group was evidence of positive change. Studies demonstrating negative or no change in attitude scores were categorized as “no positive change” (not successful). All study variables were categorical, specifically intervention type (course/rotation/course and rotation/mentoring), duration of intervention (short/medium/long), focus of questionnaire (older adults in general/older patients), fully reported response rates (yes/no), use of validated questionnaire (yes/no), study quality (adequate/poor), intervention content (empathy building/knowledge building), and attitude change results (positive change/no positive change). Results were analyzed using chi-square tests (two-sided) unless expected cell counts were lower than 5; in the latter cases, Fisher exact tests (two-sided) were used. *P* <. 05 was considered statistically significant.

**Table 1:** Intervention Studies’ Attitude Changes According to Study Characteristics

	Positive Change, n = 14	No Positive Change, n = 13
Characteristic	n	n
Type of intervention		
Rotation	2	4
Course	5	5
Course and rotation	4	2
Mentoring	3	2
Duration of intervention		
Short (<2 weeks)	5	5
Medium (2 weeks to 6 months)	4	2
Long (>6 months)	5	6
Participants		
1st- and 2nd-year medical students	6	6
3rd-, 4th-, and 5th-year medical students	6	4
All years	0	1
Doctors only	1	2
Data missing	1	0
Groups		
No comparison group	8	7
Comparison group	6	6
*i) Randomized to groups*	*1*	*3*
*ii) Not randomized to groups*	*5*	*3*
Focus of questionnaire		
Older adults in general	9	6
Older patients	5	7
Methodological quality		
Poor	4	3
Acceptable	10	10
Response rate		
Fully reported	8	5
Not fully reported	6	8
Validity statistics of questionnaire		
Known	10	7
Unknown	4	6
Intervention content		
Knowledge-building	3^a^	8^a^
*i) Methodologically poor studies removed*	*2*	*7*
*ii) Studies using unvalidated questionnaires removed*	*0*	*5*
*iii) both i) and ii) above removed*	*0*	*4*
Empathy-building	11^a^	5^a^
*i) Methodologically poor studies removed*	*8*	*3*
*ii) Studies using unvalidated questionnaires removed*	10	2
*iii) both i) and ii) above removed*	*7*	*1*

a p = .03.

## Results

Twenty-seven studies met the eligibility criteria for the present review.^6^–^32^ The main points from each study are presented in Table 2. Studies were explored in terms of their quality, design, and findings.

**Table 2:** Characteristics of Included Studies

Authors	Demographics	Study Design	Intervention	Intervention Category and Details	Attitude Assessment	Claimed Changes
Baum & Nelson (2007)^6^	67 1st-year internal medicine residents, United States	Single group pre–posttest	New geriatrics long-term care rotation, 12 months	Knowledge-building	Maxwell-Sullivan Attitude Survey (Maxwell & Sullivan, 1980)	Positive
Bernard et al. (2003)^7^	225 1st- and 2nd-year medical students, United States	Pre–posttest with comparison group	Healthy Seniors mentorship program, intermittent over 2 years (experimental) vs no exposure (comparison) over 1 year	Empathy-building (mentoring)	Aging Semantic Differential (Rosencranz & McNevin, 1969)	Positive
Carmel et al. (1992)^8^	47 1st-year medical students, Israel	Single group pre–posttest with follow-up	Geriatrics course, intermittent, 25 hours in total, over approximately 1 year	Empathy-building (informal contact)	Locally developed	No change
Deary et al. (1993)^9^	133 4th- and 5th-year medical students, United Kingdom	Separate sample pre–posttest	Geriatrics course with attached geriatric rotation, 4 weeks	Knowledge-building	Locally developed	Positive
Diachun et al. (2006)^10^	42 1st-year medical students, Canada	Posttest and follow-up with comparison group	Geriatrics course, experiential learning (intervention) vs didactic learning (comparison), 3 hours	Empathy-building (aging simulation)	Modified Palmore bias score from Facts on Aging (Palmore, 1977)	No change
Diachun et al. (2010)^11^	262 3rd-year medical students, Canada	Pre–posttest with comparison group	Geriatrics rotation (experimental) vs nongeriatric rotation (comparison), 2 weeks	Knowledge-building	Modified UCLA Geriatric Attitudes Scale (Reuben et al., 1998)	No change
Duke et al. (2009)^12^	71 1st-year medical students, United States	Single group pre–posttest	Seniors mentoring program, intermittent over 1 year	Empathy-building (mentoring)	Modified UCLA Geriatric Attitudes Scale (Reuben et al., 1998)	Positive
Eskildsen & Flacker (2009)^13^	129 1st-year medical students, United States	Single group pre–posttest	Geriatric course, 1 week	Empathy-building (informal contact)	UCLA Geriatric Attitudes Scale (Reuben et al., 1998)	Positive
Fields et al. (1992)^14^	127 4th-year medical students, United States.	Single group pre–posttest	Geriatrics rotation, 4 weeks	Knowledge-building	Aging Semantic Differential (Rosencranz & McNevin, 1969)	No change
Gonzales et al. (2010)^15^	208 1st and 2nd-year medical students, United States	Pre–posttest with comparison group	Healthy Seniors mentorship program, four 2-hour sessions (experimental) vs no exposure (comparison) over 1 year	Empathy-building (mentoring)	Refined Aging Semantic Differential (Polizzi, 2003)	Positive
Hughes et al. (2008)^16^	70 4th-year medical students, United Kingdom.	Single group pre–posttest	Geriatric course incorporating clinical training, 8 days	Knowledge-building	Modified UCLA Geriatric Attitudes Scale (Reuben et al., 1998)	No change
Intrieri et al. (1993)^17^	96 3rd-year medical students, United States	Pre–posttest with comparison group	Psychiatry clinical rotation with gerontology training program (experimental) vs same rotation without gerontology program (comparison), 6 weeks	Empathy-building (aging simulation)	Aging Semantic Differential (Rosencranz & McNevin, 1969)	Positive
Lee et al. (2005)^18^	61 geriatrics fellows, United States	Single group pre–posttest	Geriatric medicine fellowship training, 1 year	Knowledge-building	UCLA Geriatrics Attitudes Scale (Reuben et al., 1998)	No change
Lindberg & Sullivan (1996)^19^	93 internal medicine residents (PGY1, PGY2, PGY3), United States	Multiple treatment groups with random assignment, pre–posttest	Geriatrics rotation with attending geriatrician (full experimental) vs same rotation without attending geriatrician (quasi-experimental) vs no exposure to rotation (comparison), 4 weeks	Knowledge-building	Modified Maxwell-Sullivan Attitude Survey (Maxwell & Sullivan, 1980)	No change
Linn & Zeppa (1987)^20^	179 3rd-year medical students, United States	Single group pre–posttest	Surgical rotation, 12 weeks	Knowledge-building	Locally developed	Positive
Lorraine et al. (1998)^21^	100 4th-year medical students, United States	Single group pre–posttest	Aging simulation workshop (3 hours) as part of geriatrics clerkship, 2 weeks	Empathy-building (aging simulation)	Aging Semantic Differential (Rosencranz & McNevin, 1969)	Positive
Lu et al. (2010)^22^	137 1st-year medical students, United States	Pre–posttest with comparison group	Healthy Seniors mentorship program (experimental) vs no exposure (comparison), 1 year	Empathy-building (mentoring)	Aging Semantic Differential (Rosencranz & McNevin, 1969)	No change
MacKnight & Powell (2001)^23^	83 1st-year medical students, Canada	Single group pre–posttest	Geriatrics course, 6 hours over approximately 1 week	Empathy-building (informal contact)	Form A of the Opinions about People questionnaire (Ontario Welfare Council Aging Section, 1971)	Negative
Neiman et al. (1992)^24^	105 2nd-year medical students, United States	Single group pre–posttest	Geriatrics course, intermittent over 2 semesters	Knowledge-building	Locally developed	No change
Pacala et al. (1995)^25^	55 4th-year medical students, United States	Pre–posttest with comparison group	Aging simulation workshop (experimental) vs no exposure (comparison), 3 hours	Empathy-building (aging simulation)	Aging Semantic Differential (Rosencranz &McNevin, 1969); modified Maxwell-Sullivan Attitude Scale (Maxwell & Sullivan, 1980); locally developed empathy measure	Positive
Shue et al. (2005)^26^	161 1st-year medical students, United States	Pre–posttest with comparison group (posttest only)	Healthy older adults mentorship program, at least 14 1-hour visits over 1 year.	Empathy-building (mentoring)	Modified Maxwell-Sullivan Attitude Scale (Maxwell & Sullivan, 1980)	No change
Stewart et al. (2007)^27^	At least 277 medical students (year group cannot be determined), United States	Multiple treatment groups, pre–posttest (and additional follow-up for one group)	New medical school curriculum, groups received partial treatment over 2 years (quasi experimental—cohorts 1 & 2) vs full treatment over 4 years (experimental—cohorts 3 & 4)	Empathy-building (informal contact)	Modified Aging Semantic Differential (Rosencranz & McNevin, 1969)	Positive
Van Zuilen et al. (2001)^28^	288 3rd- and 4th-year medical students, United States	Single group pre–posttest	Geriatrics course including rotation, 2 weeks	Knowledge-building	Palmore bias score from Facts on Aging Quiz (1977) and Facts on Aging Quiz II (1981)	Negative
Warren et al. (1983)^29^	80 3rd-year medical students, United States	Single group pre–posttest	Geriatrics training program, including Geriatrics rotation, 6 weeks	Empathy-building (aging simulation)	Locally developed	Positive
Wilkinson et al. (2002)^30^	186 2nd-year medical students, New Zealand	Pre–posttest with comparison group	Community contact program, allocated to older adults (experimental groups) or younger adults (comparison), 1 week	Empathy-building (informal contact)	Modified Aging Semantic Differential (Polizzi & Steitz, 1998)	Positive
Wilson & Glamser (1982)^31^	82 1st-year medical students, United States	Single group pre–posttest	Geriatrics course, 2 days over 2 weeks	Empathy-building (informal contact)	Aging Semantic Differential, (Rosencranz & McNevin, 1969)	Positive
Zwahlen et al. (2010)^32^	347 1st-, 2nd-, 3rd-, 4th-, and 5th-year medical students, United States	Single group, pretest, partial-exposure, and posttest	New medical school curriculum: full exposure of 2 years (experimental), partial exposure after 1 year (quasi- experimental), and pre-implementation (comparison)	Knowledge-building	UCLA Geriatric Attitudes Scale (Reuben et al., 1998)	No change

PGY = postgraduate year; UCLA = University of California at Los Angeles.

### Design and Quality of Studies

A number of methodological weaknesses were revealed that threatened the internal validity of reported findings. For example, 15 of the 27 studies did not employ a comparison group. Without data from a comparison group, it cannot be safely concluded that changes in attitude scores were not the result of effects such as maturation (naturally occurring psychological processes during the interval between test administrations), testing (the influence of taking the test multiple times), or history (events occurring outside of the intervention during the interval between test administrations).

Seven of the 27 studies demonstrated other methodological weaknesses that might have compromised results.^6^,^7^,^10^,^16^,^23^,^25^,^27^ Examples of these problems included the use of paired statistical tests on data that were not exclusively paired,^6^ conducting more than 20 *t*-tests on the same data set without correcting the family-wise error rate,^23^–^25^ comparing a 2-year intervention group with changes in a comparison group over 1 year,^7^ high attrition levels,^10^,^16^ and significantly altering the response format of a previously established measure without piloting beforehand.^27^

There were significant problems with the validity of attitude measures. The Maxwell-Sullivan Attitude Survey was never formally validated.^33^ Palmore’s Bias scores derive from the author’s Facts on Aging Questionnaires,^34^,^35^ which are measures of knowledge about older adults, not measure of attitudes. In addition, the Aging Semantic Differential^36^ was designed for and validated in a general population rather than using healthcare professionals. Only the University of California at Los Angeles *(UCLA) Geriatrics Attitude Scale,^37^ which was employed in six studies,^11^–^13^,^16^,^18^,^32^ has been validated on a sample of healthcare professionals. Five studies used a locally developed questionnaire to measure attitudes and did not report any details on the validity of the measure.^8^,^9^,^20^,^24^,^29^ Overall, 10 of the 27 studies used a measure that had never reported details of its validity statistics in any population.^6^,^8^–^10^,^19^,^20^,^24^,^26^,^28^,^29^

Many studies failed to discriminate between attitudes toward older adults in general and older patients. As a result, studies frequently contained a mismatch between the focus of the intervention, which addressed older patients, and the terminology and focus of the questionnaire, which addressed older adults in general. Of the established measures, only the UCLA Geriatric Attitudes Scale^37^ and the Maxwell-Sullivan Attitudes Survey^38^ measure attitudes toward older patients.

### Findings Related to Attitude Change

An overview of all 27 studies that attempted to change attitudes toward older adults demonstrated inconsistent results; 14 were effective and 13 were not. Fisher exact tests revealed that positive change in attitudes was not associated with intervention type (*P* = .71, Fisher exact test), duration of intervention (*P* = .79, Fisher exact test), focus of the questionnaire (*P* = .45, chi-square), whether response rates were fully reported (*P* = .45, chi-square), whether a validated questionnaire measure was used (*P* = .44, Fisher exact test), or methodological quality of the study (*P* = .55, Fisher exact test). Positive attitude change was associated only with the intervention content (*P* = .03, chi-square) whether the intervention included an empathy-building component, in addition to any knowledge-building, or on its own. Studies containing knowledge- and empathy-building components did not show significantly different patterns from studies consisting solely of empathy building (*P* = .50, Fisher exact test). Therefore, studies were categorized into two groups: those that were focused on knowledge-building only and those that endeavored to foster empathy.

Knowledge-building interventions often consisted of lectures on geriatrics topics or clinical attachments in older patient care and typically focused on medical diagnosis and treatment. Empathy-building interventions encouraged participants to relate to or share experiences with older adults outside the medical setting. Such interventions included aging simulation exercises, designed to enable a participant to experience the difficulties and frustrations that may come with aging. Participants might wear gloves to simulate tactile deficits or earplugs to simulate hearing loss.^39^ Contact with a healthy older adult was also considered to be empathy building, whether it was a single session or series of sessions (mentoring), because these sessions involved talking to, or listening to the experiences of, an older adult to learn about their day-to-day life.

When interventions included an empathy-building component, 11 of 16 studies indicated positive attitude change. When only knowledge-building was involved, only three of 11 studies indicated positive attitude change.

## Discussion

Assessment of study quality highlighted a need for better controlled investigations, with 15 of the 27 studies not using a comparison group to ascertain attitude change. A number of studies demonstrated a mismatch between the target of the intervention (older patients) and the population evaluated in the questionnaire measure (older adults in general). Older patients are a subgroup within the older adult population and by definition are ill or unwell. The differences between the two groups have been largely overlooked in many studies. It is plausible that attitudes toward ill older adults (patients) may differ from attitudes toward healthy older adults. Questionnaires measuring attitudes toward older adults in general also tended to have a broad focus and often included items irrelevant to the patient and healthcare context (e.g., “Neighborhoods where the elderly predominate often become run down”^29^). It is recommended that future research seek to ensure that the attitude measure and the intervention focus are consistent with one another (older patients or older adults in general).

The intervention studies reviewed varied greatly in terms of design and quality. Of the methodological factors investigated, only intervention content indicated a relationship with the success or failure of an intervention to improve attitude scores. Studies that consisted solely of knowledge-building interventions were ineffective at changing attitudes. Studies that included an empathy-building task as part or all of the intervention were more likely to result in positive attitude change after the intervention. This pattern of results was evident when all studies, whatever their quality, were included. This pattern of results became even clearer when studies with major methodological weaknesses and those that used unvalidated attitude questionnaires were excluded. Variations in study quality and the use of unvalidated questionnaires may have obscured overall patterns of attitude change results in the literature. Future studies should use validated instruments to measure attitudes to allow greater confidence that they adequately capture the phenomenon under investigation.

All 27 studies claimed to include an intervention designed to improve attitudes, and 11 employed interventions with only geriatrics knowledge-building content. This knowledge-building content tended to involve training medical students and doctors in subjects relevant to care of older patients, such as providing information about common geriatric diagnoses and treatment in the older patient population. This review found that teaching medical students and doctors about the care of older patients did not appear to result in positive attitude change. In contrast, studies that included an empathy-building task—on its own or in addition to knowledge-building content—were associated with greater likelihood of positive attitude change. Empathy-building tasks encouraged participants to encounter older adults directly or listen to the experiences of older adults or attempted to simulate the experience of being older using materials designed to mimic the changes that can occur to the body as an individual grows older. In all of the empathy-building tasks, participants were encouraged to hear about or experience what it feels like to be an older adult. In some studies, the empathy-building tasks included contact with healthy older adults. The findings of this review suggest that the inclusion of an empathy-building component in a geriatrics-based intervention may increase the chances of finding postintervention positive attitude change.

## Limitations

A large number of search terms can be used to describe medical students and doctors, and it is possible that, in this review, some terms were not identified. Despite best efforts to locate articles, some may have been inadvertently missed. Although efforts were made to assess and extract data systematically, the judgments made for this review were subjective, and other researchers might have come to different conclusions. The overall sample of 27 studies was small, which was a limitation of the present review. This study employed one reviewer, and interrater reliability was not assessed. Additional reviewers might have come to different decisions, especially with regard to the inclusion of studies and judgments of study methodological quality. Moreover, because of publication bias, studies finding nonsignificant results are less likely to be published than those with significant findings, which may have skewed the results of the present review. Therefore studies showing no change in attitudes after a geriatrics intervention may be underrepresented in the present review. This review sought to identify studies of medical students and doctors working in secondary and tertiary care settings and so did not include primary care physicians or those working in community-based practices.

## Conclusion

In conclusion, we propose that enabling a medical student or doctor to interact with older adults or consider how it may feel to be an older adult may be more likely to result in positive attitude change than an educational intervention, even if that intervention is designed to make them more knowledgeable with regard to older patient care. Future geriatrics interventions should seek to corroborate these findings by including an empathy-building component.
